# Diagnostic test accuracy of PCR by saliva specimen for cytomegalovirus infection in newborn: A protocol for systematic review and meta-analysis

**DOI:** 10.1097/MD.0000000000031776

**Published:** 2022-11-25

**Authors:** Bo Zheng, Fang Fang Wu, Xiao Xiao Li, Ren Shen, Zong Zheng, Hong Yan Liu

**Affiliations:** a Yuhuan People’s Hospital, Taizhou, China; b Ruian People’s Hospital, Wenzhou, China.

**Keywords:** cytomegalovirus, meta-analysis, newborn, PCR, rapid culture, saliva, sensitivity, specificity, urine

## Abstract

This study aimed to assess the diagnostic accuracy of polymerase-chain-reaction by saliva.

**Method:**

We searched Pubmed, EMBASE, Cochrane Library and Web of Science. A case–control or cohort study designed to explore the saliva specimens for diagnosing the cytomegalovirus infection was eligible for this study. Sensitivity (SEN), specificity (SPE), diagnostic odds ratio (DOR), and summary receiver operating characteristic curves were calculated.

**Results:**

Nine articles were selected for the meta-analysis. For assessing saliva PCR with urine culture, the SEN, SPE, and DOR were 0.97 [95% confidential interval (CI):0.61,1.00] 1.00 [95%CI:0.99, 1.00] 15672 [95%CI:558, 440325], respectively; for assessing saliva PCR with urine PCR, the SEN, SPE, and DOR were 0.87 [95%CI:0.79, 0.92] 1.00 [95%CI:1.00, 1.00] 15637 [95%CI:5946, 41126], respectively; for total assessment, the SEN, SPE, and DOR were0.91 [95%CI:0.70, 0.98] 1.00 [95%CI:1.00, 1.00] 8493 [95%CI:1429, 50487], respectively.

**Conclusion:**

We reported that saliva PCR performed well in the diagnosis of cytomegalovirus infection.

## 1. Introduction

Remarkable improvements in the management of cytomegalovirus (CMV) infection have been achieved and urine samples are the most widely used samples for CMV infection diagnosis among newborns.^[1]^ However, the identification of a congenitally infected newborn infant soon after birth before hospital discharge is still hard to achieve as urine samples are hard to obtain. Some studies used saliva analysis may represent an easier, more practical, and less expensive approach.^[2,3]^ However, much of the uncertainty related to the diagnostic accuracy of saliva samples from studies with small sample sizes and heterogeneous methodologies, which make it difficult to draw firm conclusions. Therefore, this study aimed to examine the diagnostic accuracy of PCR using saliva samples for newborns with cytomegalovirus infection.

## 2. Methods

### 2.1. Overview

Our study protocol, which has been registered in the International Prospective Register of Systematic Reviews (CRD42022337717) [Bo Z, Fang Fang W, Xiao Xiao L, et al. Diagnostic test accuracy of PCR by saliva specimen for cytomegalovirus infection in newborn: an international prospective register of systematic reviews], and the Cochrane handbook and diagnostic accuracy study reviews were used to guide the analysis.^[4,5]^ The study followed a prior established protocol.

### 2.2. Eligibility criteria

#### 2.2.1. *Type of studies*.

We had planned to include both case-control studies and cohort studies as well as prospective and retrospective studies, as long as they could provide sufficient data concerning both the sensitivity and specificity of the CMV PCR assay when using PCR or rapid culture as the reference standard.^[6,7]^ Conference abstracts and reports written in languages other than English were excluded. Studies that only covered sensitivity or specificity were excluded. Nonhuman studies, such as those on murine CMV, were also excluded.

#### 2.2.2. *Participants*.

We assessed subjects for all Infants within 4 weeks.

### 2.3. *Index and reference test*

The index test was a CMV polymerase chain reaction (PCR) assay using saliva samples. The reference tests were CMV PCR by urine,^[8–11]^ rapid culture by urine,^[2,12,13]^ rapid culture by saliva,^[3]^ and 1 study used PCR and culture by urine as the reference test.^[14]^ PCR tests included nested PCR and RT-PCR. Some of the tests did not mention the type of PCR that was performed.^[2,3,11]^ Two studies used nested PCR.^[10,12]^ Four studies used RT-PCR.,^[8,9,13,14]^ 1 study used 2 genes(i.e., and gB) when testing by RT-PCR,^[13]^ and 1 study used the gB gene when testing by nested PCR.^[12]^ Other 7 studies did not mention the gene type.

### 2.4. Literature search strategy

In the electronic search, we systematically searched PubMed, EMBASE, the Cochrane Library, and Web of Science on July 06, 2022. Two investigators independently screened the candidate articles by checking the title and abstract after uploading the citation list into ENDNOTE 20.4 (Bld.18004). After independent screening, articles that were still regarded as candidates by at least 1 investigator were then independently scrutinized through full-text reading. The final inclusion was decided after resolving discrepancies between the investigators.

### 2.5. Search strategy

The following descriptors were included: “infant, newborn” [MeSH] and “CMV” [Title/Abstract] and “saliva” [Title/Abstract], simplified. The full descriptor is on this site [The search strategy used in Diagnostic test accuracy of PCR by saliva specimen for cytomegalovirus infection in newborn: systematic review and meta-analysis]. The bibliographies of the included studies and recent review articles were also searched to identify additional studies.

### 2.6. Study selection and data extraction

Data were extracted by 2 reviewers independently and then cross-checked. The following data were extracted: baseline study characteristics – primary author, title of publication, year of publication, geographical location of patient population, and study design; specimen type – saliva sample; diagnostic test – PCR by saliva and; the proportion of positive and negative results for each diagnostic test.

The data were also cross-checked again by additional reviewers. If 2 assays were evaluated as the standard references, a rapid culture of urine was preferred.^[2,8,14]^ Meta-analysis was conducted based on the assumption that each specimen was independent from another specimen. Inputting the data from 1 specimen twice was not allowed, because this would lead to duplicate use of the same data.

Study quality was determined using the Quality Assessment of Diagnostic Accuracy Studies 2 (QUADAS-2) tool.^[15–17]^ We used the standard 4 QUADAS-2 domains: patient selection, index test, reference standard, and flow/timing. The patient population was defined as a newborn within 4 weeks, and the index test was defined as a saliva-based PCR test. The reference test was defined as a saliva-based culture test or a urine-based test, and both PCR and culture were accepted. The overall risk of bias was evaluated for all 4 domains. The flow/timing domain was excluded from the applicability analysis. Each study was assigned an overall judgment of “low,” “high,” or “unclear” risk by both authors, and all discrepancies were resolved by consensus. For sensitivity analysis, a study with at least 1 domain scored as high risk or high applicability concern was regarded as a high-risk study.

### 2.7. Statistical analysis

Review Manager 5.3^[16]^ and Stata 17 were used for statistical analysis. Sensitivity and specificity were calculated for each available comparison. The main outcomes were the diagnostic test accuracy of PCR-diagnosed CMV infection in saliva evaluated by the following statistics: diagnostic odds ratio (DOR), area under the curve (AUC), and summary estimates of the sensitivity (SEN), specificity (SPE), positive likelihood ratio (PLR), and negative likelihood ratio (NLR).

We used both the hierarchical summary receiver operating characteristics model and bivariate model. Heterogeneity, which is the degree of inconsistency among studies, was assessed using the I2 statistic:0% to 40% represents not important heterogeneity, 30% to 60% represents moderate heterogeneity, 50% to 90% represents substantial heterogeneity, 75% to 100% indicates considerable heterogeneity. To determine overall accuracy, we calculated the DOR and AUC values. DOR is a measure of the effectiveness of a diagnostic test, wherein DOR = 1 means no diagnostic value, DOR > 1 means that a test positive suggests disease positivity, and DOR < 1 means that a test negative suggests disease positivity. We obtained a paired forest plot, hierarchical summary receiver operating characteristic curve, and summary estimates of sensitivity and specificity using the bivariate model. PLR and NLR were calculated from summary estimates of sensitivity and specificity. AUC, PLR, and NLR were interpreted according to the 4-grade criteria.

The AUC indicates how accurate a test is: an AUC in the ranges of < 0.75, 0.75 to 0.92, 0.93 to 0.96, and > 0.97 meant “not accurate good,” “very good” and “excellent,” respectively. PLR defined by “sensitivity/ (1-specifificity)” and NLR defined by “(1 -sensitivity)/ specificity” represent how the test results change the probability of a disease. PLR values in the range of < 2, 2 to 5, 5 to 10 and > 10 were recognized as showing a “not meaningful,” “small,” “moderate” and “large” increase in probability, respectively. NLR in the range of > 0.5, 0.2 to 0.5, 0.1 to 0.2, and < 0.1 represented a “not meaningful,” “small,” “moderate” and large’ decrease of probability, respectively.

### 2.8. Subgroup and sensitivity analyses

As a subgroup analysis, we focused on studies that exclusively evaluated PCR by saliva versus PCR by urine,^[8–11]^ PCR by saliva versus culture by urine or saliva.^[2,3,12–14]^ Another subgroup analysis we focused on 5 prospective studies.^[3,10–12,14]^ However, the data from retrospective studies^[8,13]^ were not enough to be analyzed. Remaining 2 studies did not mention the studying type.^[2,9]^

### 2.9. Publication bias

Publication bias was assessed graphically by Funnel plots and the Deek’s Funnel Plot Asymmetry test *P* < .5 was defined as a publication bias positive.

### 2.10 . Trial sequential analysis

Trial sequential analysis was assessed graphically to ensure whether the sample size is enough to generate a significant result.

## 3. Results

### 3.1. Study search results and characteristics

**Figure 1.**
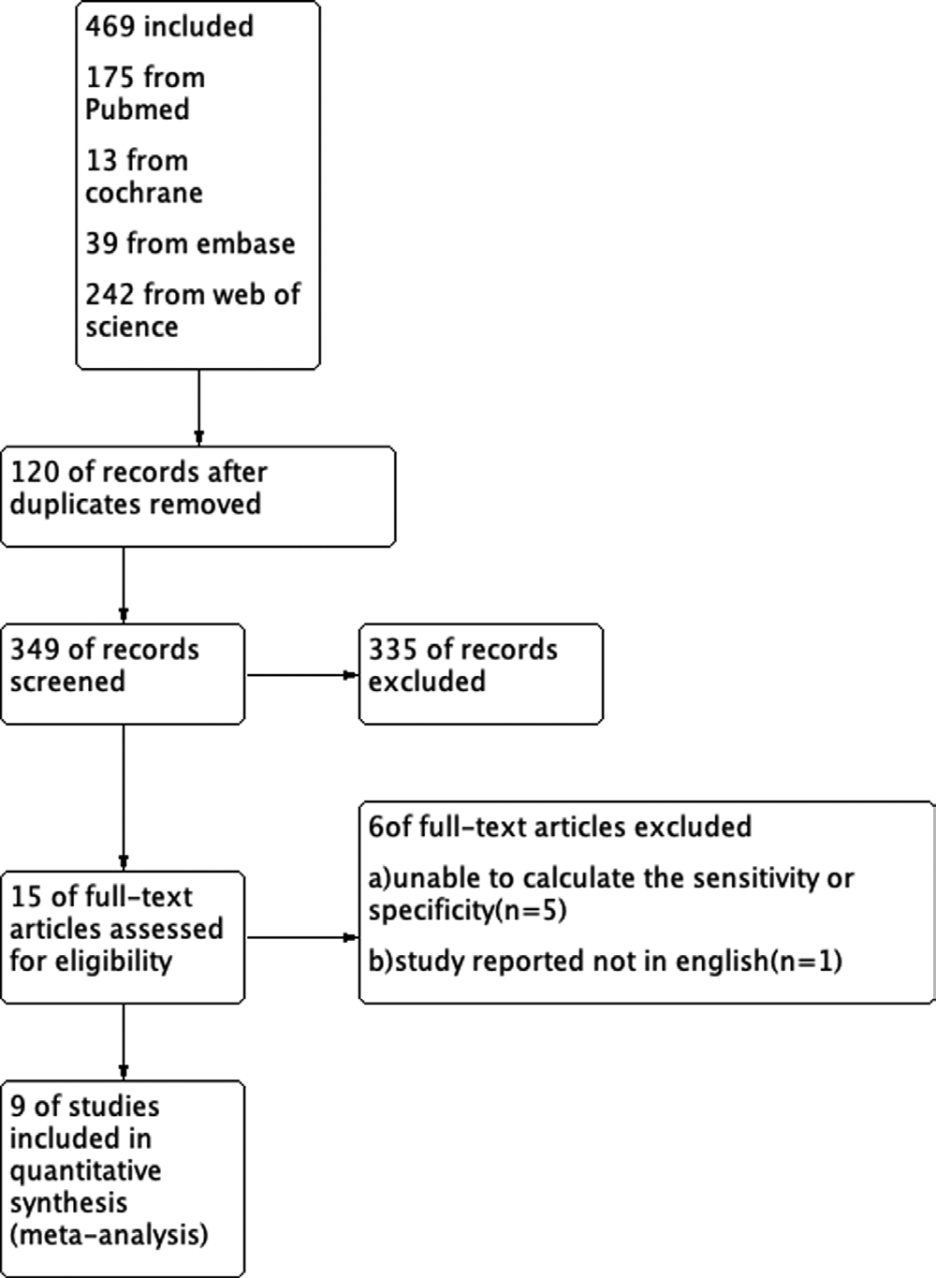
The preferred reporting items in systematic reviews and meta-analyses flow chart for study search.

**Figure 2.**
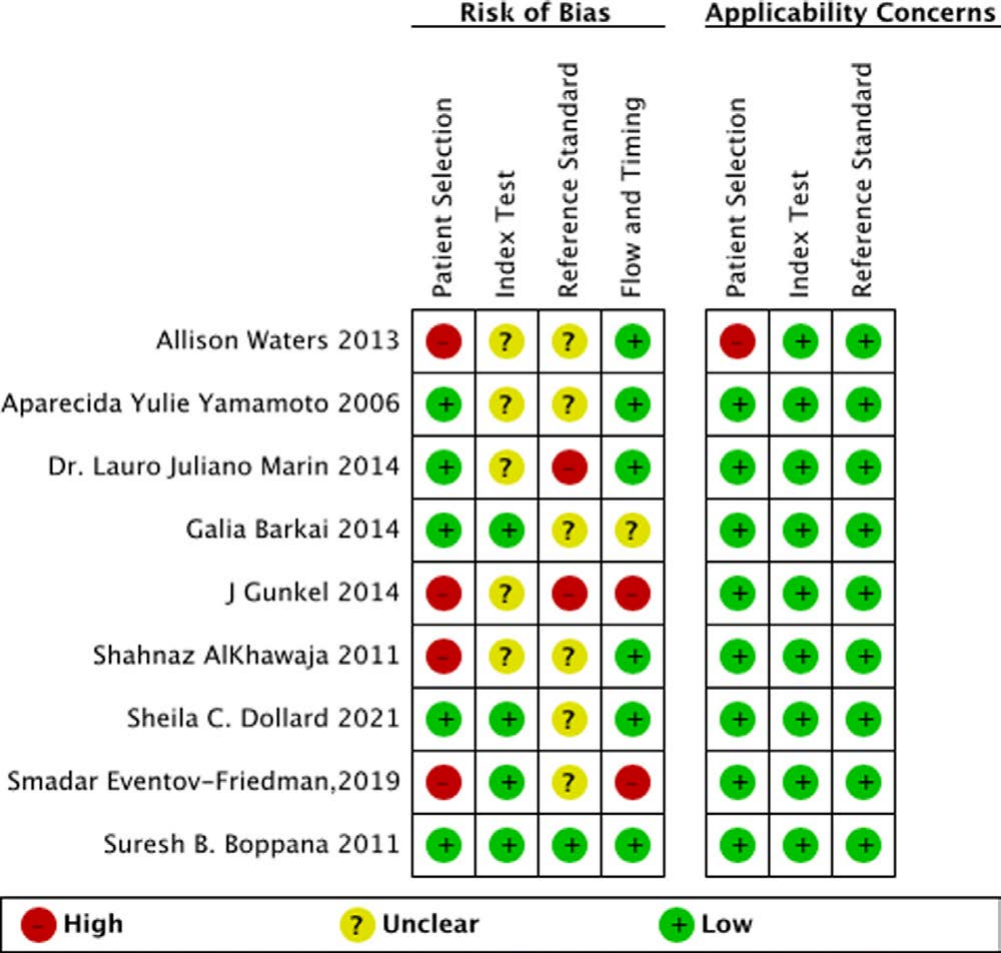
Methodological quality summary

**Figure 3.**
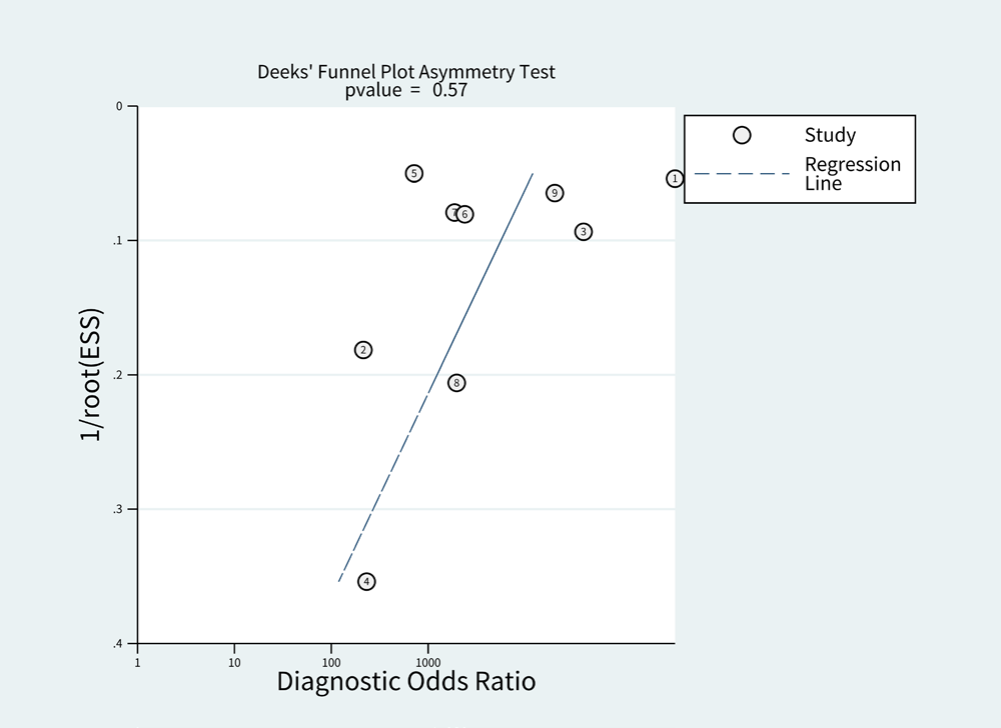
Publication bias of the meta-analysis

A total of 469 citations were identified in the initial database search. Based on the abstract review, 15 studies were selected for full-length manuscript evaluation. After applying the inclusion and exclusion criteria, 9 citations were included.^[2,3,8–14]^ Figure 1 depicts the study flow. The Deek’s Funnel Plot Asymmetry test *P* = .57which is > 0.5, so there is no publication bias, as shown in Figure 2. The sample sizes were enough for this meta-analysis, as trial sequential analysis had shown graphically in Figure 3.

The study characteristics are summarized in Table 1. Of the studies that were published from 2006 to 2021, 9 studies were full-length manuscripts and 5 studies were prospective,^[3,10–12,14]^ and 2 studies were retrospective.^[3,8]^ and 2 studies were unclear.^[2,9]^ All studies included PCR data from saliva specimens, of which 2 studies also included culture data from saliva.^[3,12]^ Two studies were case-control.,^[2,12]^ and the other 7 studies were cohort-studies.^[3,8–11,13,14]^ Two studies excluded symptomatic newborns,^[8,12]^ while other studies included all newborns. Three studies did not mention about the type of PCR that was performed.^[2,3,11]^ Two of the studies used pooled-samples^[8,12]^, and other studies did not mention about it.

**Table 1:** Patient and Study Characteristics of Eligible Studies Assessing PCR by saliva.

study	Study type a	Case no b	Study location	Age	Type of test *c*	Type of test *d*
Boppana, S. B. (2011)	CS, P	17,662	US	<4wk	liquid-and dried-saliva PCR	rapid culture of saliva
Alkhawaja, S. (2012)	CC, P	153	Arab	<3wk	nested PCR (gB)	shell vial culture (urine or saliva)
Yamamoto, A. Y. (2006)	CC, U	1923	Brazil	<3wk	PCR	Culture of urine
Waters.A (2014)	CS, R	1044	Ireland	<1wk	Rt-PCR	Rt-PCR of urine
Eventov-Friedman.S (2019)	CS, P	856	Israel	<2wk	Rt-PCR	culture of urine
Barkai.G (2014)	CS, R	235	Israel	<2wk	rt-PCR (i.e., and gB genes)	Culture of urine
Gunkel, J. (2014)	CS, U	261	Netherlands	<3wk	Rt-PCR	Rt-PCR in urine
Cardoso, E. S. (2015)	CS, P	333	Brazil	<3wk	nested PCR in saliva	nested PCR in urine
Dollard, S. C. (2021)	CS, P	12,544	US	<2wk	PCR	PCR in urine

a CC = case–control, CS = cohort-study, P = prospective, R = retrospective. U = unclear.

b no = number, c for test group, d for group of reference standard.

PCR = polymerase chain reaction; rt PCR = real-time polymerase chain reaction.

### 3.2. Meta-analysis of all the studies

Used data from all 9 studies, consisting of 398 PCR-positive subjects and 34622 PCR-negative subjects, the DOR was 8493 [95% confidential interval 1429, 50487; *I*2 = 95.95%], and AUC was 1.00 [0.99–1.00].

The summary estimates of sensitivity specificity PLR, and NLR were 0.91 [0.70, 0.98] 1.00[1.00, 1.00] 797.5[206.2,3084.4] and0.09 [0.03, 0.34], respectively. I2 was 95.55% indicating considerable heterogeneity, and subgroups were considered to reduce the heterogeneity.

### 3.3. Subgroup and sensitivity analyses

**Figure 4.**
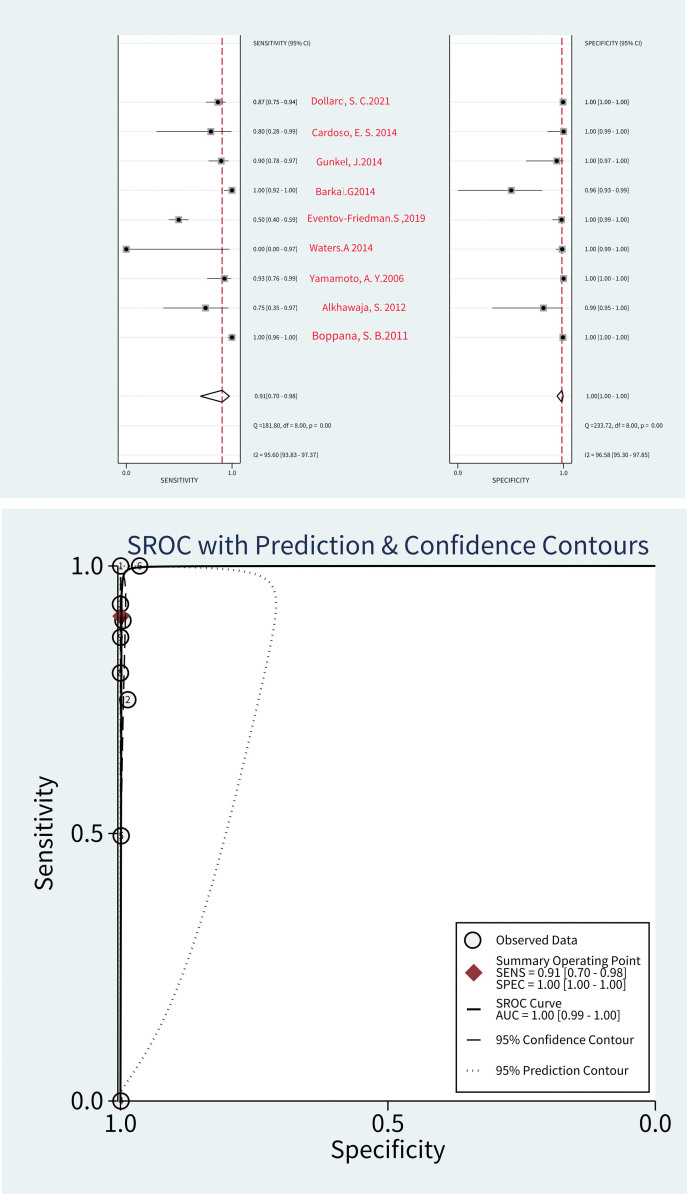
Results of diagnostic meta-analysis, including the pooled sensitivity, specificity, and SROC curve. SROC = summary receiver operating characteristic

**Figure 5.**
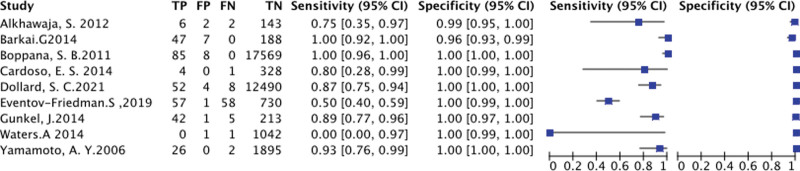
A paired forest plot for the diagnosis accuracy of PCR by saliva for cytomegalovirus in newborn. TP = true positive; FP = false positive; FN = false negative; TN = true negative.

The results of the subgroup analyses based on prospective studies are shown in Table 2. The DOR based on prospective studies was 8310[725,95281; *I*2 = 73%]. The AUC based on them was 1.00 [0.99–1.00], as shown in Figure 4 and Figure 5.

**Table 2:** Patient and study characteristics of eligible studies assessing saliva PCR in CMV infection for newborns.

	All	prospective	PCR^ a^	Culture ^b^
Study	35,020	31,512	14,194	20,826
PCR positive	398	273	115	283
PCR negative	34,622	31,275	14,079	20,543
DOR	8493[1429, 50,487] *I*2 = 96%	8310[725, 95,281] *I*2 = 73%	15,637[5946,41126] *I*2 = 100%	15,672[558, 4,40,325] *I*2 = 95%
AUC	1.00 [0.99–1.00]	1.00 [0.99–1.00]	1.00 [0.99–1.00]	1.00 [0.99–1.00]
SEN	0.91 [0.70, 0.98]	0.88 [0.55,0.98]	0.87 [0.79, 0.92]	0.97 [0.61, 1.00]
SPE	1.00[1.00, 1.00]	1.00[1.00, 1.00]	1.00[1.00, 1.00]	1.00[0.99, 1.00]
PLR	797.5[206.2,3084.4]	1028.0[278.1,3799.4]	2040.5[914.0,4555.5]	548.1[70.2,4277.4]
NLR	0.09 [0.03, 0.34]	0.12[0.03, 0.57]	0.13[0.08, 0.21]	0.03 [0.00, 0.56]

a: saliva PCR versus urine PCR.

b: saliva PCR versus culture by urine or saliva.

CMV = cytomegalovirus, DOR = diagnostic odds ratios, NLR = negative likelihood ratios, PLR = positive likelihood ratios, SEN = sensitivity, SPE = specificity.

Studies assessing saliva PCR with urine culture or saliva culture are also shown in Table 2. The DOR compared saliva PCR with urine PCR and the DOR compared saliva PCR with culture by urine or saliva were 15,637[5946, 41,126; *I*2 = 100%] and 15672[558, 44,0325; *I*2 = 95%], respectively. The AUC based on these values were 1.00 [0.99–1.00] and 1.00 [0.99–1.00], respectively. Their sensitivities based on them were 0.87 [0.79, 0.92] and 0.97 [0.61, 1.00], respectively. their specificities were 1.00 [1.00, 1.00] and 1.00 [0.99, 1.00], respectively. Studies assessing saliva PCR with culture by urine or saliva showed a high level of sensitivity, as shown in Figure 4 and Figure 5.

**Figure 6.**
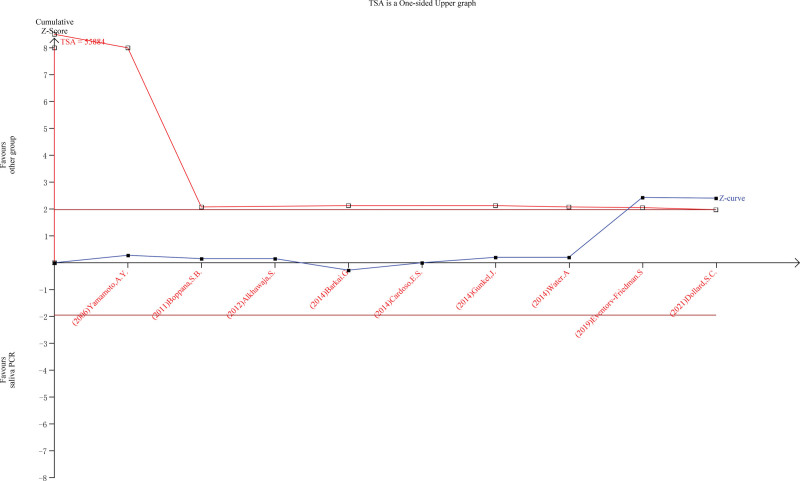
Methodological quality summary.

Sensitivity analyses using data from studies without high risk were performed.^[2,3,11,13]^ which are shown in Figure 6. The results of these sensitivity analyses did not differ significantly from those from all the studies. The AUC were 1.00 [0.99–1.00], the DOR were 113498 [4426, 29,10,622; *I*2 = 95%], the sensitivity were 0.99 [0.65, 1.00], the specificity were 1.00 [0.99, 1.00].

## 4. Discussion

Our systematic review detected 9 original studies that compared results from saliva PCR assay with urine PCR assay, urine culture assay and saliva culture assay. The sensitivity and specificity of the saliva PCR were consistently high, and the summary estimates of sensitivity and specificity were 0.91 [0.70, 0.98] and 1.00[1.00, 1.00]. According to the subgroup analyses, when saliva PCR was compared with urine culture, the sensitivity and specificity were 0.97 [0.61, 1.00] and 1.00[0.99, 1.00]. We do have reason to recommend the use of PCR assay by saliva for the diagnosis of CMV infection.

The major advantage of the PCR assay over the saliva assay is that it is easy to obtain, so that more infants with CMV infection will be identified by means of clinical examination during the newborn period. The robustness of our analysis is supported by numerous factors, such as the large number of included studies and subjects, and consistent results from many sensitivity analyses.

Despite these strengths, we must comment on the limitations of our analyses. First was the definition of our gold standard, various standards are used as reference standard like saliva culture, urine PCR, and urine culture. Secondly, it is likely that variability existed between institutions with respect to the number of participants. Finally, 4 study was deemed high-risk for bias based on the QUADAS- 2 assessment, the risk of bias was high. All of which cause a considerable heterogeneity.

In conclusion, we conducted the first systematic review and meta-analysis to assess the diagnostic accuracy of PCR using saliva sample. It has good sensitivity and specificity. Therefore, It is a useful diagnostic tool for CMV infection in newborns.

## Author contributions

**Conceptualization:** Fang Fang Wu.

**Data curation:** Bo Zheng, Xiao Xiao Li.

**Formal analysis:** Xiao Xiao Li.

**Investigation:** Fang Fang Wu.

**Methodology:** Bo Zheng, Ren Shen.

**Project administration:** Bo Zheng, Ren Shen.

**Resources:** Ren Shen, Zong Zheng.

**Software:** Zong Zheng.

**Supervision:** Bo Zheng, Hong Yan Liu.

**Validation:** Hong Yan Liu.

**Visualization:** Fang Fang Wu, Hong Yan Liu.

**Writing – original draft:** Bo Zheng.

**Writing – review & editing:** Bo Zheng.
